# Delayed Gastric Emptying after Living Donor Hepatectomy for Liver Transplantation

**DOI:** 10.1155/2014/582183

**Published:** 2014-12-25

**Authors:** Hanjay Wang, Adam D. Griesemer, Ronald F. Parsons, Jay A. Graham, Jean C. Emond, Benjamin Samstein

**Affiliations:** ^1^Center for Liver Disease and Transplantation, Department of Surgery, Columbia University Medical Center, New York, NY 10032, USA; ^2^Department of Surgery, Emory University School of Medicine, Atlanta, GA 30322, USA; ^3^Department of Surgery, Montefiore Medical Center, Bronx, NY 10467, USA; ^4^New York Presbyterian Hospital/Columbia, PH Room Floor 14, Room 202, 622 West 168th Street, New York, NY 10032, USA

## Abstract

Delayed gastric emptying is a significant postoperative complication of living donor hepatectomy for liver transplantation and may require endoscopic or surgical intervention in severe cases. Although the mechanism of posthepatectomy delayed gastric emptying remains unknown, vagal nerve injury during intraoperative dissection and adhesion formation postoperatively between the stomach and cut liver surface are possible explanations. Here, we present the first reported case of delayed gastric emptying following fully laparoscopic hepatectomy for living donor liver transplantation. Additionally, we also present a case in which symptoms developed after open right hepatectomy, but for which dissection for left hepatectomy was first performed. Through our experience and these two specific cases, we favor a neurovascular etiology for delayed gastric emptying after hepatectomy.

## 1. Introduction

Delayed gastric emptying (DGE) complicates living donor hepatectomy for liver transplantation in 2% of patients and is almost exclusively associated with left-sided hepatectomy [1, 2]. Symptoms include nausea, vomiting, bloating, and epigastric discomfort, which typically resolve with conservative treatment, but severe cases may require endoscopic or surgical intervention. Because living donors are generally healthy prior to surgery, postoperative DGE is an important complication to recognize, treat, and prevent. Understanding the mechanism of posthepatectomy DGE is therefore essential.

Here, we present two cases of DGE that developed after hepatectomy for living donor liver transplantation, including the first reported example of this complication following laparoscopic hepatectomy. Importantly, we also present a case in which DGE developed after an open right hepatectomy, but for which dissection for a left hepatectomy was first performed. All patient data was reviewed under IRB-approved study protocol IRB-AAAM5707. Through our experience, we favor a neurovascular etiology as the primary cause of DGE after hepatectomy.

## 2. Case 1

**Figure 1:**
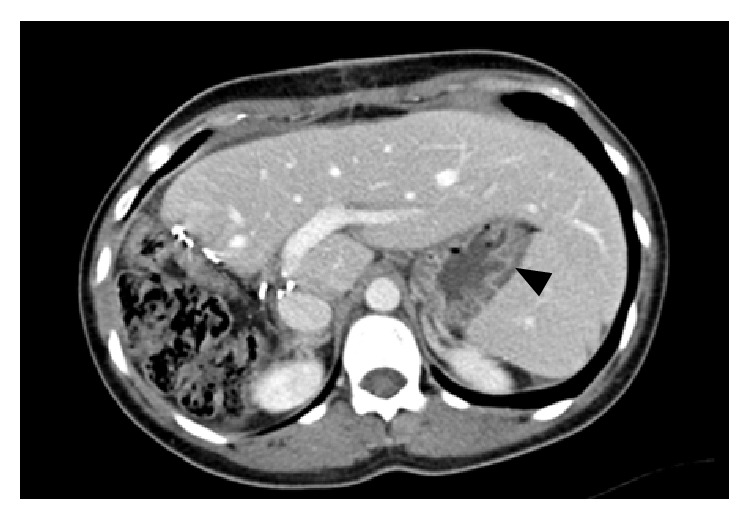
Computed tomography scan of the abdomen with intravenous contrast for patient KS, one month following open right hepatectomy. There is no evidence of adhesion formation between the stomach and cut liver surface. The stomach (black arrow) is far removed from the cut surface of the liver.

**Figure 2:**
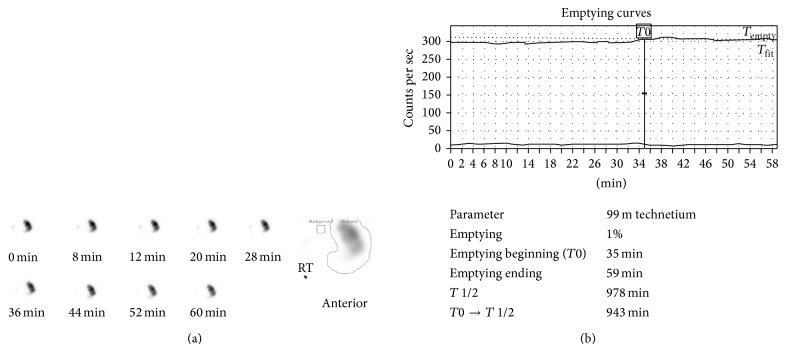
Gastric emptying study for patient KS following open right hepatectomy. (a) Gastric emptying was observed for 60 minutes with intermittent imaging. The region of interest is indicated at right by a line drawn around the activity within the stomach in the anterior view. (b) The emptying curves over 60 minutes (*T*_empty_—solid; *T*_fit_—dotted) are shown. The half-emptying time (*T* 1/2) was calculated using the geometric mean. Gastric emptying time was markedly prolonged, with absent emptying during the 60-minute period.

**Figure 3:**
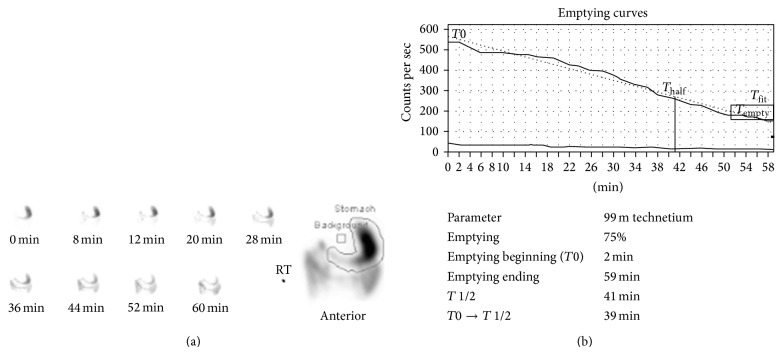
Gastric emptying study for patient KS following intrapyloric botulinum toxin injection. (a) Gastric emptying was observed for 60 minutes with intermittent imaging. The region of interest is indicated at right by a line drawn around the activity within the stomach in the anterior view. (b) The emptying curves over 60 minutes (*T*_empty_—solid; *T*_fit_—dotted) are shown. The half-emptying time (*T* 1/2) was calculated using the geometric mean. Gastric emptying time was normal with 75% emptying over 60 minutes and a half-emptying time of 41 minutes.

KS, a 31-year-old woman with mild gastroesophageal reflux disease requiring no home medications, presented for planned open left hepatectomy (our preferred technique at the time) for liver donation. During the procedure, a replaced left hepatic artery was visualized and dissected to the level of its origin from the left gastric artery. To completely mobilize the replaced artery, neurovascular branches supplying the lesser curvature of the stomach were divided. Upon examination of the hepatic hilum, a standard left hepatic artery was found, as well as a small segment-4 artery from the proximal right hepatic artery. These additional arteries were not seen on preoperative imaging. Due to concern for increased risk of graft failure, the decision was made to convert to a right hepatectomy. The operation proceeded without complications. After removal of the right lobe graft, omentum and colon were placed in the resection bed, and Seprafilm adhesion barrier was applied to the cut liver surface. During the first postoperative week, the patient complained of nausea, heartburn, reflux, and nonbilious vomiting. The patient gradually improved with medical management including a proton pump inhibitor, sucralfate, and metoclopramide, and she was discharged home on postoperative day (POD) 9. On POD 31, the patient was readmitted for severe nausea, bilious vomiting, and burning epigastric pain. A computed tomography (CT) scan of the abdomen did not suggest bowel obstruction or adhesion formation between the stomach and cut liver surface (Figure 1). Endoscopy revealed retained food products in the stomach, and a gastric emptying study demonstrated absent emptying over one hour (Figure 2). Following initial improvement with medical management as described above, the patient experienced worsening emesis on POD 43 with solid food trial. During repeat endoscopy, 200 units of botulinum toxin were injected at the pylorus. Afterward, the patient experienced no further emesis and tolerated oral medications. An upper gastrointestinal series with small bowel follow-through showed no abnormalities. The patient was discharged home on POD 49. She underwent a repeat gastric emptying study one week after discharge, which showed marked improvement and normal gastric emptying (Figure 3). In subsequent follow-up, she reports feeling well and eating normally. She is currently over four years postdonation.

## 3. Case 2

**Figure 4:**
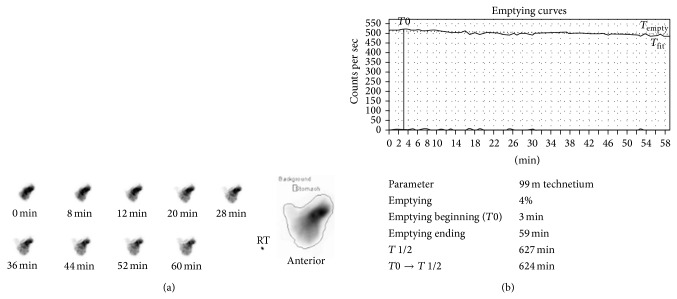
Gastric emptying study for patient IS following laparoscopic left hepatectomy. (a) Gastric emptying was observed for 60 minutes with intermittent imaging. The region of interest is indicated at right by a line drawn around the activity within the stomach in the anterior view. (b) The emptying curves over 60 minutes (*T*_empty_—solid; *T*_fit_—dotted) are shown. The half-emptying time (*T* 1/2) was calculated using the geometric mean. Gastric emptying time was markedly delayed, with no definitive evidence of radiotracer passing into the duodenum during the 60-minute period.

**Figure 5:**
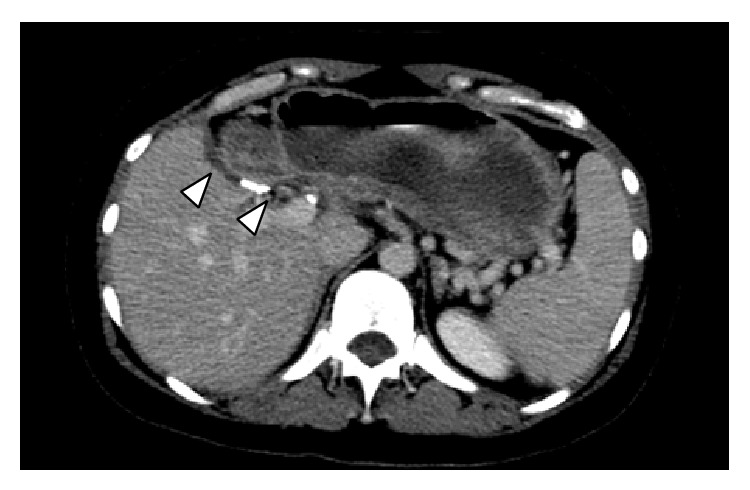
Computed tomography scan of the abdomen with intravenous contrast for patient IS, two weeks following laparoscopic left hepatectomy. There is no evidence of a structural etiology for delayed gastric emptying, including adhesion formation between the stomach and cut liver surface (white arrows).

IS, a healthy 46-year-old woman, underwent a totally laparoscopic full left hepatectomy for liver donation, using our previously described technique [3]. A replaced left hepatic artery was visualized during the procedure. To have a larger arterial orifice, branches to the lesser curvature of the stomach were taken and the artery was divided at the level of the left gastric artery. The operation proceeded without complications. The patient had a postoperative course notable for mild heartburn and bloating, controlled with a proton pump inhibitor and H2 receptor antagonist, and was discharged home on POD 6. On POD 10, the patient was readmitted for nausea, epigastric pain, and heartburn. A gastric emptying study demonstrated marked delay suggesting gastroparesis (Figure 4). Endoscopy revealed a deformity of the gastric antrum and food residue within the stomach. 40 units of botulinum toxin were injected into the deformed area of the stomach, and the patient reported prompt improvement of her symptoms. A subsequent abdominal CT scan showed passage of contrast through the small bowel. In addition, there was no evidence on CT scan to suggest a structural etiology for DGE, such as adhesion formation between the stomach and cut liver surface (Figure 5). The patient was discharged home on POD 18. Currently, she is over 6 months postdonation and reports feeling well and eating normally but continues to take a proton pump inhibitor for heartburn.

## 4. Discussion

Postoperative DGE occurs in 2% of all living liver donors and is primarily associated with left-sided hepatectomy [1, 2], although Kim et al. reported six cases (1.2%) in a series of 500 right-sided liver donors [4]. The mechanism of posthepatectomy DGE remains unknown. One hypothesis involves adhesion formation between the stomach and cut liver surface. After a left-sided hepatectomy, the stomach may twist and fall into the resection bed. As a result, the dislocated stomach may contact the raw surface of the remaining liver and ultimately become fixed via adhesions. Gastric obstruction may develop due to disruption of motility at the points of adhesion, leading to symptoms of DGE.

Direct visualization of gastrohepatic adhesions after left-sided hepatectomy for living liver donation has been documented via surgical reexploration in two cases by Umeshita et al. [1] and one by Kinoshita et al. [5]. All three patients reportedly experienced resolution of symptoms after lysis of adhesions. In addition, some studies have suggested that wrapping the liver with greater omentum [5] or suturing the greater omentum to the peritoneum [6, 7] may decrease the risk of posthepatectomy DGE by preventing contact between the stomach and cut liver surface. Several other studies, however, found no significant effect using the same and/or different techniques to prevent adhesions [8–12]. Overall, the majority of these studies are small, single-center, nonrandomized trials, and the collective data is ambiguous.

The cases of KS and IS add insight to our understanding of posthepatectomy DGE. To our knowledge, the case of IS represents the first report of DGE following laparoscopic hepatectomy for living donor liver transplantation. Laparoscopic abdominal surgeries generally result in fewer adhesions compared to open procedures [13]. Indeed, one study involving pigs found that, compared to open resection of the hepatic left lateral segment, laparoscopic resection resulted in a marked decrease in postoperative adhesions [14]. Despite not using an adhesion barrier in the case of IS, we did not find evidence of postoperative adhesions between the stomach and cut liver surface on CT scan.

The case of KS is particularly noteworthy in the fact that we initially performed the dissection for a left hepatectomy but ultimately used a right lobe graft. As a result, the resection bed was far removed from the stomach, and the cut liver surface was faced away from the stomach. The absence of apposition significantly decreases the probability of adhesion formation between the stomach and the cut surface of the liver. Indeed, we did not find evidence of postoperative gastrohepatic adhesions on CT scan for KS.

An alternative hypothesis for the cause of posthepatectomy DGE involves injury to branches of the anterior vagal trunk coursing through the gastrohepatic ligament, which is typically divided during dissection and mobilization of the liver [15]. In particular, injury to the nerves of Latarjet, the vagal efferents that innervate the gastric antrum and pylorus, may compromise antral contractility and pyloric relaxation, ultimately resulting in delayed emptying and retention [16]. Symptom onset during the immediate postoperative period after hepatectomy is consistent with a neurovascular etiology. Moreover, in the cases of both KS and IS, the injection of botulinum toxin into the pylorus promptly led to symptom resolution. Botulinum toxin, which blocks the release of acetylcholine into neuronal synapses, decreases pyloric muscle activity when injected locally and has been reported to reduce symptoms in gastroparesis patients [17]. The efficacy of intrapyloric botulinum toxin injection in the cases of KS and IS indicates that impaired pyloric relaxation may contribute to posthepatectomy DGE, and our observation is consistent with the possibility that injury to vagal nerve branches innervating the pylorus may be the cause.

Notably, both of our cases involved a replaced left hepatic artery, an anatomical variant in which the left hepatic artery arises not from the proper hepatic artery as in normal anatomy but from the left gastric artery. In the context of performing a left hepatectomy for liver donation, a replaced left hepatic artery requires surgeons to perform a more extensive dissection of the gastrohepatic ligament in order to prepare the arterial graft from the left gastric artery. Greater dissection within this area increases the risk of vagal nerve injury and may therefore increase the risk of DGE. To our knowledge, the relationship between replaced vascular anatomy and risk of DGE after hepatectomy has not yet been investigated, but several groups have previously suggested that increased manipulation of the hepatic hilum may result in higher risk of DGE after surgery [8, 9, 18]. The presence of a replaced left hepatic artery in both cases of KS and IS required us to thoroughly dissect the gastrohepatic ligament and take down neurovascular branches to the lesser curvature of the stomach, a process that may have contributed to the onset of DGE in the immediate postoperative period.

Overall, based on our experience and specifically the cases of KS and IS, we favor the hypothesis that extensive dissection of the gastrohepatic ligament may increase the risk of posthepatectomy DGE due to increased risk of damage to vagal branches in the area. Further studies involving larger case collections and multicenter experiences are required to elucidate the true mechanism underlying the development of DGE after living donor hepatectomy.
